# Spatial and Sexual Divergence of Gut Bacterial Communities in Field Cricket *Teleogryllus occipitalis* (Orthoptera: Gryllidae)

**DOI:** 10.1007/s00248-023-02265-z

**Published:** 2023-07-21

**Authors:** Kazuya Hirata, Toru Asahi, Kosuke Kataoka

**Affiliations:** 1 Graduate School of Advanced Science and Engineering, Waseda University, Tokyo, Japan; 2 Comprehensive Research Organization, Waseda University, Tokyo, Japan; 3 Research Organization for Nano & Life Innovation, Waseda University, Tokyo, Japan

**Keywords:** Gut microbiome, 16S rRNA sequencing, Field cricket, *Teleogryllus occipitalis*

## Abstract

The insect gut is colonized by microbes that confer a myriad of beneficial services to the host, including nutritional support, immune enhancement, and even influence behavior. Insect gut microbes show dynamic changes due to the gut compartments, sex, and seasonal and geographic influences. Crickets are omnivorous hemimetabolous insects that have sex-specific roles, such as males producing chirping sounds for communication and exhibiting fighting behavior. However, limited information is available on their gut bacterial communities, hampering studies on functional compartmentalization of the gut and sex-specific roles of the gut microbiota in omnivorous insects. Here, we report a metagenomic analysis of the gut bacteriome of the field cricket *Teleogryllus occipitalis* using 16S rRNA V3-V4 amplicon sequencing to identify sex- and compartment-dependent influences on its diversity and function. The structure of the gut microbiota is strongly influenced by their gut compartments rather than sex. The species richness and diversity analyses revealed large difference in the bacterial communities between the gut compartments while minor differences were observed between the sexes. Analysis of relative abundance and predicted functions revealed that nitrogen- and oxygen-dependent metabolism and amino acid turnover were subjected to functional compartmentalization in the gut. Comparisons between the sexes revealed differences in the gut microbiota, reflecting efficiency in energy use, including glycolytic and carbohydrate metabolism, suggesting a possible involvement in egg production in females. This study provides insights into the gut compartment dependent and sex-specific roles of host-gut symbiont interactions in crickets and the industrial production of crickets.

**Supplementary Information:**

The online version contains supplementary material available at 10.1007/s00248-023-02265-z.

## Introduction

The insect gut is a desirable, nutrient-rich ecological niche for multiple microorganisms. Insect guts are colonized by symbiotic bacteria that can regulate a variety of host processes, such as the digestion of food to improve growth and development [1], compensation for nutrient-deficient diets [2], the immune system [3], mate choice, and reproductive efficiency [4, 5].

Insect gut is highly compartmentalized and has distinct functions. It generally consists of three regions: foregut, midgut, and hindgut. These regions are functionally and structurally specialized, reflecting adaptations to different niches and feeding habitats of insects [6–10]. The foregut and hindgut are directly connected to the mouth and anus, respectively, with the foregut structurally specialized for temporal food storage and the hindgut for reabsorbing nutrients and water and holding feces before defecation. The midgut is the main region for nutrient digestion and absorption; it lacks an exoskeletal lining, unlike the other regions, and secretes a protective envelope known as the peritrophic matrix in many insect species [6–8, 10]. Malpighian tubules in the anterior region of the hindgut excrete waste products, including nitrogen and other solutes, into the hindgut to provide nutrients, creating a different nutritive environment for the microbial communities [6–8]. Such structural and functional specializations in the insect gut subregions have led to the presence of unique microorganisms in specific gut compartments [11, 12].

There is also evidence of sex-biased variance in the insect gut microbiome. This variance can be shaped by sex-specific nutritional requirements, physiology, and behavior. For example, females need sources of proteins and lipids to fulfill their reproductive potential, as reported in beetles [13] and crickets [14]. Several studies using beetles [15], black soldier flies [16], and mosquitoes [17] have demonstrated that sex plays different roles in bacterial composition and abundance in the gut and frass. Mating also influences the female gut microbiota structure in the Mormon cricket *Anabrus simplex* (Orthoptera: Tettiginiidae) [18]. Although much research has investigated the diversity of the gut microbiota in insects, few studies have focused on sexual differences influencing gut microbial communities.

Crickets have been studied as a useful model for hemimetabolous insects. Crickets have recently gained important societal value as a novel alternative protein source [19]. Previous studies have reported that *Acheta domesticus* (Orthoptera: Gryllidae), which possesses gut bacteria, digests water-soluble plant polysaccharides more efficiently than gut bacteria-free insects, suggesting that bacteria colonizing the insect hindgut may be responsible for this digestive ability [20]. In *Gryllus pennsylvanicus* (Orthoptera: Gryllidae), gut bacteria differentially influence food selection between sexes [14], raising the possibility that gut bacterial communities exhibit sexual differentiation in crickets. Recent advancement of next-generation sequencing technology has enabled more comprehensive and detailed studies of the gut microbiota, including uncultured bacteria. The gut microbiome structure of house cricket *Acheta domesticus* and Jamaican field cricket *Gryllus assimilis* has recently been identified at high resolutions [21]. Furthermore, the gut microbiome of the Mormon cricket *Anabrus simplex* (Orthoptera: Tettiginiidae) was also reported to greatly vary across different regions of the gut [11]. Hence, we can infer that the gut microbiome of crickets may differ between different gut compartments or sexes, thus conferring a compartmentalized or sexually dimorphic effect on the host. However, no studies have compared the differences in the gut microbiota of crickets between gut compartments or sexes.

Here, we characterized the gut microbial communities of the field cricket *Teleogryllus occipitalis* (Orthoptera: Gryllidae), focusing on individual gut compartments and sex differences, using deep sequencing of the 16S rRNA V3-V4 region. This species is harvested from the wild or is reared as an edible insect in Asian countries. The genome datasets of this species were available in our previous work, providing the potential for studying molecular interactions between host and bacterial symbionts colonized in the gut [22]. We conducted differential relative abundance and functional prediction of the gut microbial communities of crickets in different gut compartments and sexes. Our findings will contribute to a better understanding of host-microbiome interactions and the effects of gut compartments and sex on the gut microbiome.

## Materials and Methods

### Gut Dissection and DNA Extraction

*T. occipitalis* was obtained from a population collected from Amami Oshima Island, Kagoshima, Japan. We reared *T. occipitalis* from eggs to adults at 30 °C (16 h L:8 h D). Cricket food (Tsukiyono Farm, Gunma, Japan), and water was supplemented once every 2–3 days ad libitum in the laboratory. All individuals were kept together in the same 86-L plastic cases.

**Fig. 1:**
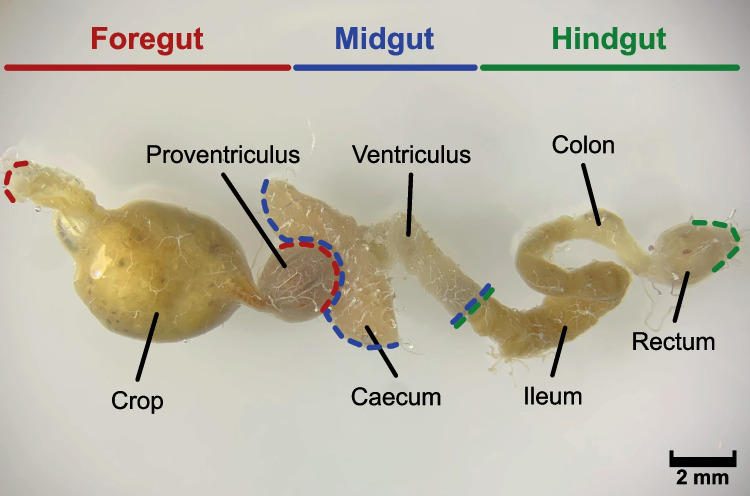
The morphology of foregut, midgut, and hindgut in *T*. *occipitalis*

Male (*n* = 9) and female (*n* = 5) crickets were surface-sterilized with 70% ethanol before the foregut (crop), midgut (ventriculus), and hindgut (ileum, colon, and rectum) were dissected using flame-sterilized tools and aseptically homogenized for each individual (Fig. 1). Genomic DNA from each gut compartment of each individual was extracted using a DNeasy Blood and Tissue Kit (QIAGEN, Valencia, CA, USA) after 30 min of lysozyme incubation to lyse Gram-positive bacterial walls, according to the manufacturer’s instructions. In addition, the genomic DNA of cricket feed was also extracted using the same method described above and analyzed in the same manner as the other samples.

### DNA Sequencing

Bacterial V3-V4 16S rRNA libraries were constructed using a two-step tailed PCR method. The first PCR was performed using the universal bacterial DNA primers V3/V4f_MIX (ACACTCTTTCCCTACACGACGCTCTTCCGATCT-NNNNN-CCTACGGGNGGCWGCAG) and V3/V4r_MIX (GTGACTGGAGTTCAGACGTGTGCTCTTCCGATCT-NNNNN-GACTACHVGGGTATCTAATCC) (341F and 805R) provided by the Bioengineering Lab. Co. Ltd. (Sagamihara, Japan). The PCR reaction was performed by KOD One PCR master mix (Toyobo, Japan) under the following conditions: initial denaturation at 98 °C for 1 min, followed by 45 cycles of denaturation at 98 °C for 10 s, annealing at 55 °C for 5 s, extension at 68 °C for 1 s, and a final extension at 68 °C for 5 min. Subsequent PCR library construction and sequencing were performed by Bioengineering Lab. Co. Ltd. PCR products were purified using AMPure XP, and a second PCR was conducted using forward (5′-AATGATACGGCGACCACCGAGATCTACAC-Index2-ACACTCTTTCCCTACACGACGC-3′) and reverse (5′-CAAGCAGAAGACGGCATACGAGAT-Index1-GTGACTGGAGTTCAGACGTGTG-3′) primers. The second PCR reaction was performed by Ex Taq HS (Takara Bio, Japan) under the following conditions: initial denaturation at 94 °C for 2 min, followed by 10 cycles of denaturation at 94 °C for 30 s, annealing at 60 °C for 30 s, extension at 72 °C for 30 s, and a final extension at 72 °C for 5 min. The purified PCR product concentration of the library using AMPure XP was checked by the Synergy H1 and QuantiFluor dsDNA Systems. The quality of the library was verified using the Fragment Analyzer and dsDNA 915 Reagent Kit (Agilent Technologies, CA, USA). The sequence was analyzed on an Illumina MiSeq instrument using a MiSeq Reagent Kit v3 kit (Illumina, CA, USA) under 2 × 300 bp conditions.

### DNA Sequencing Data Processing

Raw sequence data were processed using QIIME 2 (v. 2021.4.0) [23]. Raw read sequences were quality checked and demultiplexed by per-sample barcodes using “q2-demux summarize” function. The sequence reads were filtered with a quality score of at least 20. Denoising and chimera removal were performed using “q2-dada2 denoise-paired” function in DADA2 [24]. Taxonomy of the amplicon variant sequences (ASVs) was assigned by “q2-feature-classifier” function against the SILVA database (v. 138) [25]. Next, the ASV sequences were aligned using the “q2-alignment mafft” and “q2-alignment mask” functions, and a phylogenetic tree was constructed using “q2-phylogeny fasttree” and “q2-phylogeny midpoint-root” functions. In addition, reads assigned to the mitochondria and chloroplasts were filtered.

### Clustering Analysis

Hierarchical clustering was performed using the ggheatmap package (v. 2.20) [26] in R software [27]. The ggheatmap package computed the euclidean distance matrix for each sample with the “dist” function of the stats package (v 3.6.2) in R (method = “euclidean”), and then used the “hclust” function to perform hierarchical clustering using the complete-linkage method (method = “complete”). To exclude rare taxa, only gut bacteria from the phylum, family, and genus levels that were present in at least 30% of the total sample and had a relative abundance of at least 0.1% for each sample were included in the hierarchical clustering analysis of the bacterial composition profiles for each gut compartments and sex.

### Alpha and Beta Diversity Analysis

For alpha and beta diversity analyses, libraries were resampled to a constant depth of 9153 sequences (minimum number of sample sequences in the total sample). Alpha diversity indices (Chao1 [28], Shannon [29], and Simpson [30]) were calculated using the phyloseq package (v. 1.42.0) [31] in R. In addition, Faith’s phylogenetic diversity (PD) [32] was calculated by the “q2-diversity core-metrics-phylogenetic” function. Alpha diversities from samples with different gut compartments and sexes were first analyzed using two-way analysis of variance (ANOVA). Subsequently, multiple comparison tests of alpha diversity indices of different gut compartments were performed beginning with the Kruskal-Wallis test followed by the Wilcoxon rank sum test. For differences in alpha diversity indices between males and females, the Wilcoxon rank-sum test was performed. Beta diversity was assessed based on unweighted (species identity-based) or weighted (species abundance-based) UniFrac distances [33] via “q2-diversity core-metrics-phylogenetic” function and visualized with Principal Coordinate Analysis (PCoA). The group significance between beta diversity indices was analyzed using permutational multivariate analysis of variance (PERMANOVA).

### Differential Relative Abundance Analysis of the Bacterial Community and Functional Predictions

Differential relative abundance analysis of identified microbial taxa at the genus and family levels was performed using several statistical tools in R, including DESeq2 (v 1.34.0) [34], ALDEx2 (v 1.26.0) [35], and the ANCOMBC package (v 2.0.1) [36], as recommended in a recent benchmark paper [37, 38]. For DESeq2, library size correction was performed using the “poscounts” method. ANCOMBC was executed with zero detection-enabled structure (strc_zero=TRUE, neg_lb =FALSE). ALDEx2 was performed using the default parameters. Bacterial species with relative abundances of more than 1% were included in this analysis to detect major species variations.

Functional prediction of the samples was performed using PICRUSt2 (v 2.5.0) [39] and the MetaCyc database [40]. The accuracy of the metagenomic predictions for each sample was assessed by calculating the Nearest Sequenced Taxon Index (NSTI) provided by the PICRUSt2 software, and ASVs with the NSTI values > 2 were excluded from the output. Differential analysis of PICRUSt2 predicted pathway abundance was also performed using the statistical tools described above. *P*-values were FDR-corrected using the Benjamini-Hochberg method in ALDEx2 and DESeq2 and the Bonferroni method in ANCOMBC. Statistical significance was assessed using an adjusted *P*-value of 0.05.

## Results

### Relative Abundance of Gut Microbiota in Field Cricket *Teleogryllus occipitalis*

Genomic DNA was extracted from 42 samples, composed of foreguts (crop), midguts (ventriculus), and hindguts (ileum, colon, and rectum) dissected from the gut tracts of nine males and five females (Fig. 1). A total of 1,772,115 reads were obtained from the samples using Illumina MiSeq paired-end sequencing of the V3-V4 region of the 16S rRNA gene amplicon (mean ± SE = 42,193 ± 495 per sample). After quality control, 1,256,434 reads were retained (mean ± SE = 29,915 ± 441 per sample) and used for further analysis (Table S1). These reads were binned to 3834 ASVs, rarefaction curves of which were obtained to assess species richness and were found to reach a plateau in all samples (Fig. S1), indicating that most of the microbial diversity in each sample was fully determined. We taxonomically assigned these ASVs at various levels of classification and found that they belonged to 13 phyla, 96 families, and 154 genera.

**Fig. 2:**
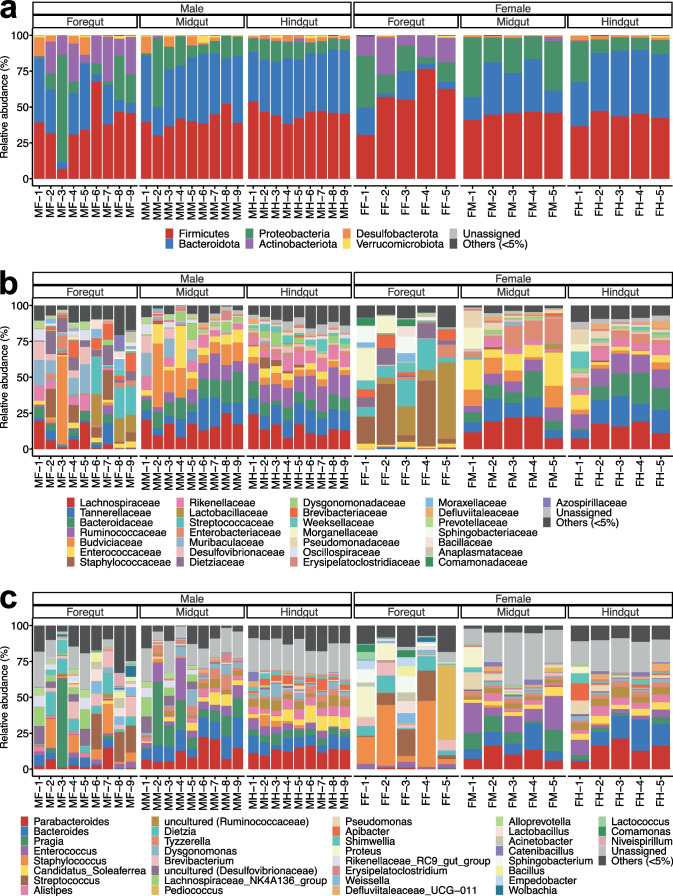
Gut bacteriome composition of *T*. *occipitalis* in each gut compartments and sex. Relative abundance of gut bacterial communities **a** at phylum level, **b** at family level, and **c** at genus level. These samples were taken from the male foregut (MF), male midgut (MM), male hindgut (MH), female foregut (FF), female midgut (FM), and female hindgut (FH)

The microbial communities of *T. occipitalis*, with the predominant phylum Firmicutes, Bacteroidota, and Proteobacteria, were almost consistent with previous studies in *T. oceanicus* (Orthoptera: Gryllidae) and the spring field cricket *Gryllus veletis* (Orthoptera: Gryllidae) (Fig. 2) [41, 42]. Several predominant families, such as Bacteroidaceae, Ruminococcaceae, and Rikenellaceae, were also found in the gut of *T. occipitalis*. However, family Porphyromonadaceae and genus *Wolbachia* were predominant in *T. oceanicus* and *G. veletis*, respectively, but not abundant in the gut microbiome of *T. occipitalis* [41, 42]. The microbial communities in the foregut at the family and genus levels varied considerably among the samples compared to those in the midgut and hindgut. The sample-to-sample variation in the foregut microbiota may be due to that the crickets were not fasted and thus there were individual differences. The relative abundances of all the identified bacterial species are shown in Table S2. In addition, the bacterial composition of the feed in this study was distinct from that of all gut compartments of the crickets (Fig. S2).

**Fig. 3:**
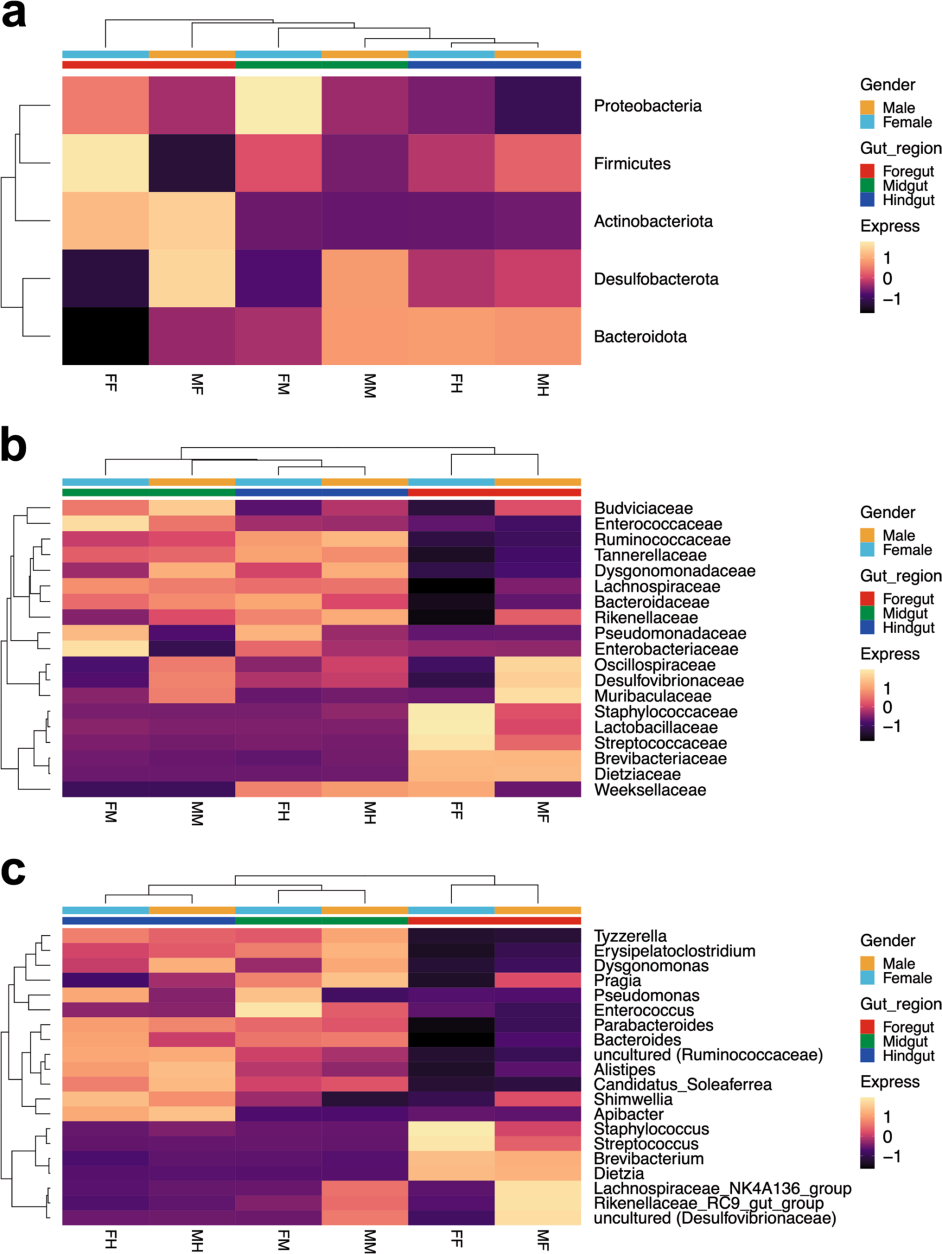
Heatmap of hierarchical clustering of the mean relative abundance of gut bacteria for each gut compartments and sex **a** at phylum level, **b** at family level, and **c** at genus level. These samples were taken from the male foregut (MF), male midgut (MM), male hindgut (MH), female foregut (FF), female midgut (FM), and female hindgut (FH)

Hierarchical clustering using Euclidean distance metrics was used to explore the relationship between the sample attributes and the composition of the gut microbiome (Fig. 3). We found that the profiles of microbial communities were clustered by gut compartments rather than by sex. At the family and genus levels, the profiles of bacterial communities from the midgut and hindgut clustered together. In contrast, the foregut microbiome did not form a cluster at any taxonomic level, which may be due to inter-sample variation.

### Diversity of Gut Microbiota

**Fig. 4:**
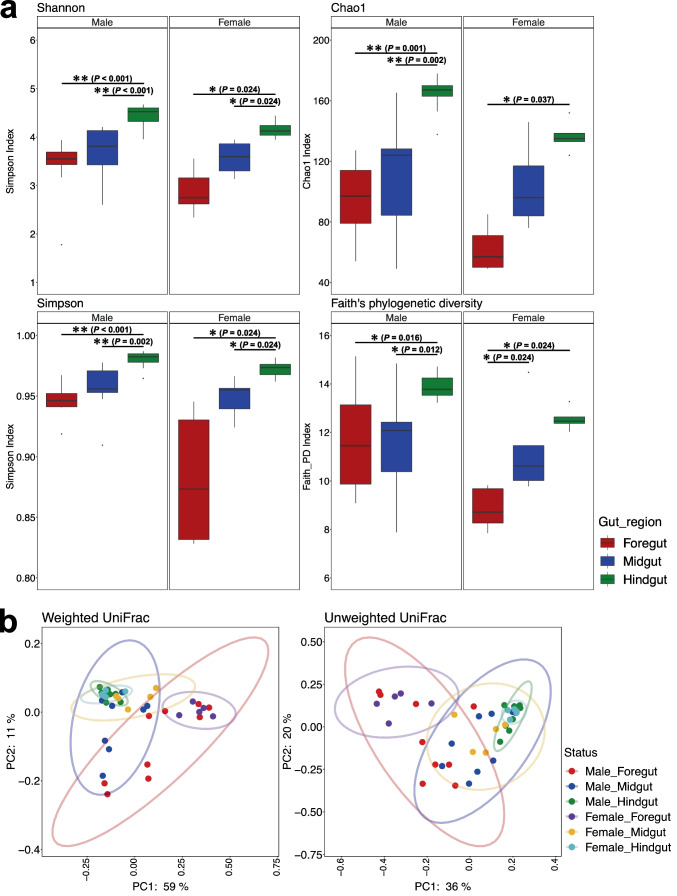
Diversity of the gut bacterial communities of *T. occipitalis* by gut compartments and sex. **a** Alpha diversity indices of gut bacterial communities, including Chao1, Shannon, Simpson, and Faith’s phylogenetic diversity (PD). The Wilcoxon rank sum test was used to compare the difference in the gut compartments. Single asterisk indicates *P* < 0.05. Double asterisk indicates *P* < 0.01. **b** Principal coordinate analysis (PCoA) with 95% confidence ellipse based on weighted UniFrac distance and unweighted UniFrac distance

Species richness and diversity indices were analyzed to characterize the gut microbial communities for each sex and region (Fig. 4a). For alpha diversity index analysis (Chao1, Shannon, Simpson, and Faith’s phylogenetic diversity (PD)), there were significant differences among gut compartments, but no differences between sexes or interactions, except for Chao1 and Faith’s PD indexes (two-way ANOVA, Table S3). Shannon and Simpson indexes in the hindguts of both males and females and Chao1 and Faith’s PD indexes in the hindguts of males were higher than those of the other gut compartments (Table S3). The Chao1 index was highest in the female hindgut followed by midgut and foregut (Table S3). The Faith’s PD index in the female foregut was lower than in the other gut compartments (Table S3). When comparing the sexes of the same gut compartments, there were significant differences in Chao1 and Faith’s PD indices in the foregut and Chao1, Shannon, and Faith’s PD indices in the hindgut, but not at all in the midgut, with the indices of males being significantly higher than those of females (Table S3, *P* < 0.05). This indicates that the foregut and hindgut of males are associated with more diverse microbial communities than those of females. We also reanalyzed the alpha diversity analysis with the singleton removed and found that the conclusions are exactly the same (Fig. S3, Table S8, *P* < 0.05).

We conducted PCoA based on unweighted and weighted UniFrac distance metrics as beta diversity. We found variable levels of individual-to-individual variation among the gut compartments (Fig. 4b). In particular, the microbial communities in the foregut varied considerably among the samples compared to those in the midgut and hindgut. PERMANOVA for both unweighted and weighted UniFrac distance metrics revealed significant differences among different gut compartments in both male and female gut microbiomes (Table S4, *P* < 0.05). In contrast, there was a significant difference only in the foregut with the unweighted UniFrac distance metric method, but there were no other significant differences in the gut microbiome between the sexes (Table S4, *P* < 0.05).

### Differential Relative Abundance Analysis Between Sexes and Gut Compartments

To identify differentially abundant taxa among different gut compartments or between sexes, a differential relative abundance analysis was performed. Samples from the foregut of both males and females were excluded from further analysis due to high sample-to-sample variation. Bacterial species with relative abundances of > 1% were included in this analysis.

First, we performed a differential analysis of the relative abundance between the midgut and the hindgut. We focused on taxa for which we detected significant differences in at least two of the three different testing tools (DESeq2, ALDEx2, and ANCOMBC). The DESeq2 method uses count data to fit a model based on a negative binomial distribution and uses a likelihood ratio test (LRT) to identify taxa with significant differences in abundance between two defined groups. ALDEx2, on the other hand, generates a Monte Carlo sample of the Dirichlet distribution using a uniform prior distribution for each sample and a CLR transformation of the estimated abundance of each bacterial species. In addition, the Wilcoxon rank test and Welch’s *t*-test are used on the transformed data to evaluate significant differences between sample groups. These methods were then corrected for false discovery rates using the Benjamini and Hochberg method. ANCOMBC uses a linear regression framework to estimate the unknown sampling fraction from the counts and normalizes the read counts in a process analogous to a log-ratio transformation. It then identifies abundant taxa that differ between the two groups. The method corrected for false discovery rates using the Bonferroni method.

**Fig. 5:**
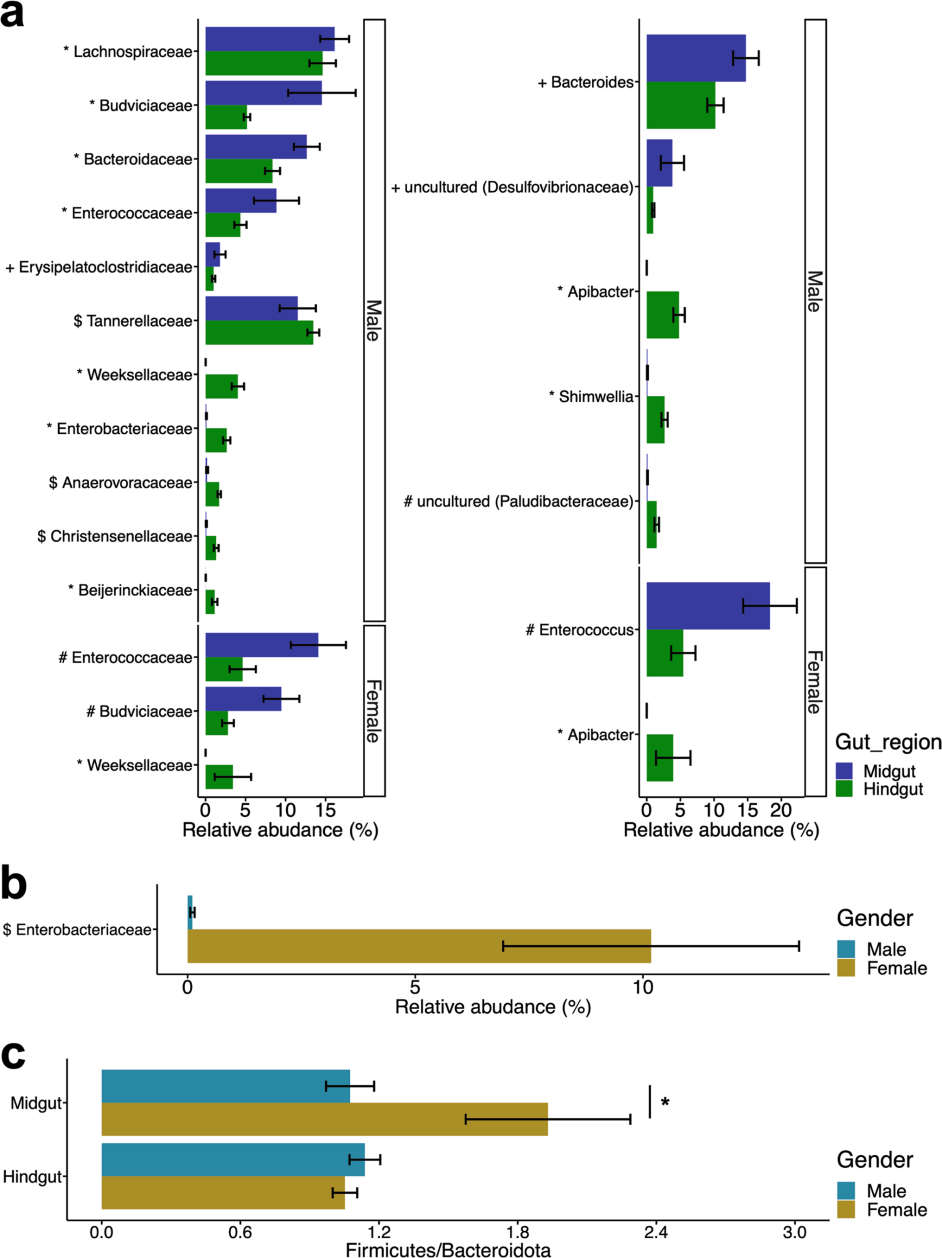
Differences in relative abundance of gut bacteria between gut compartments and sexes. Error bars indicate standard error. Relative abundance of gut bacteria with significant variation at the family and genus level among **a** gut compartments and **b** sexes. The mark before the bacterial name indicates the combination of statistical tools that detected a significant difference (*all statistical tools, #ANCOMBC and DESeq2, $ALDEx2 and ANCOMBC, +ALDEx2 and DESeq2). **c** Ratio of relative abundance of Firmicutes and Bacteroidota phylum. Asterisk (*) shows significant differences from Wilcoxon rank sum test. *P*-values were FDR-corrected using the Benjamini-Hochberg method (*P*-values < 0.05)

Comparing the midguts and hindguts of males at the family level, five families were significantly more abundant in the midgut and six families in the hindgut (Fig. 5a). In females, two families were significantly more abundant in the midgut and one in the hindgut (Fig. 5a). In both males and females, there were two families, Enterococcaceae and Budviciaceae, which were significantly more abundant in the midgut than in the hindgut (Fig. 5a). Weeksellaceae was the only family that was significantly more abundant in the hindguts of both males and females than in the midguts (Fig. 5a). Similarly, at the genus level in males, two genera (*Bacteroides* and uncultured bacteria (Desulfovibrionaceae)) in the midgut and three genera (*Apibacter*, *Shimwellia* and uncultured bacteria (Paludibacteriaceae)) in the hindgut were significantly more abundant than those in the other regions (Fig. 5a). In contrast, in females, one genus was found to be more abundant in the midgut (*Enterococcus*) or hindgut (*Apibacter*) than in other regions (Fig. 5a). There was no common genus between the sexes, which was more abundant in the midgut than in the hindgut. *Bacteroides* and uncultured bacteria (Desulfovibrionaceae) were found to be more abundant in males, whereas *Enterococcus* was found only in females (Fig. 5a). There was only one genus, *Apibacter*, in the hindgut that was abundant in both sexes (Fig. 5a). The statistical values for each tool are listed in Supplemental Tables S5, S6, and S7.

In comparisons between sexes in the midgut, Enterobacteriaceae was significantly more frequently detected in females than in males (Fig. 5b). Pseudomonadaceae and Staphylococcaceae were significantly more abundant in females only in ANCOMBC and DESeq2, respectively. In contrast, no bacterial taxa were found to be significantly different between the sexes in the hindgut. We further evaluated the Firmicutes to Bacteroidota ratio and found that the female midgut had a significantly higher ratio than that of the other groups, suggesting that the female midgut was enriched with gut bacterial communities with high glycolytic activity (Fig. 5c) [43, 44].

### Functional Prediction of Gut Microbiota with Different Sexes and Regions

We next investigated the metabolic function of cricket gut microbiota and identified 384 pathways for all samples. Differential analysis of the predicted metabolic pathways between the midgut and hindgut or between sexes was conducted using DESeq2, ALDEx2, and ANCOMBC in the same way as previously described.

**Fig. 6:**
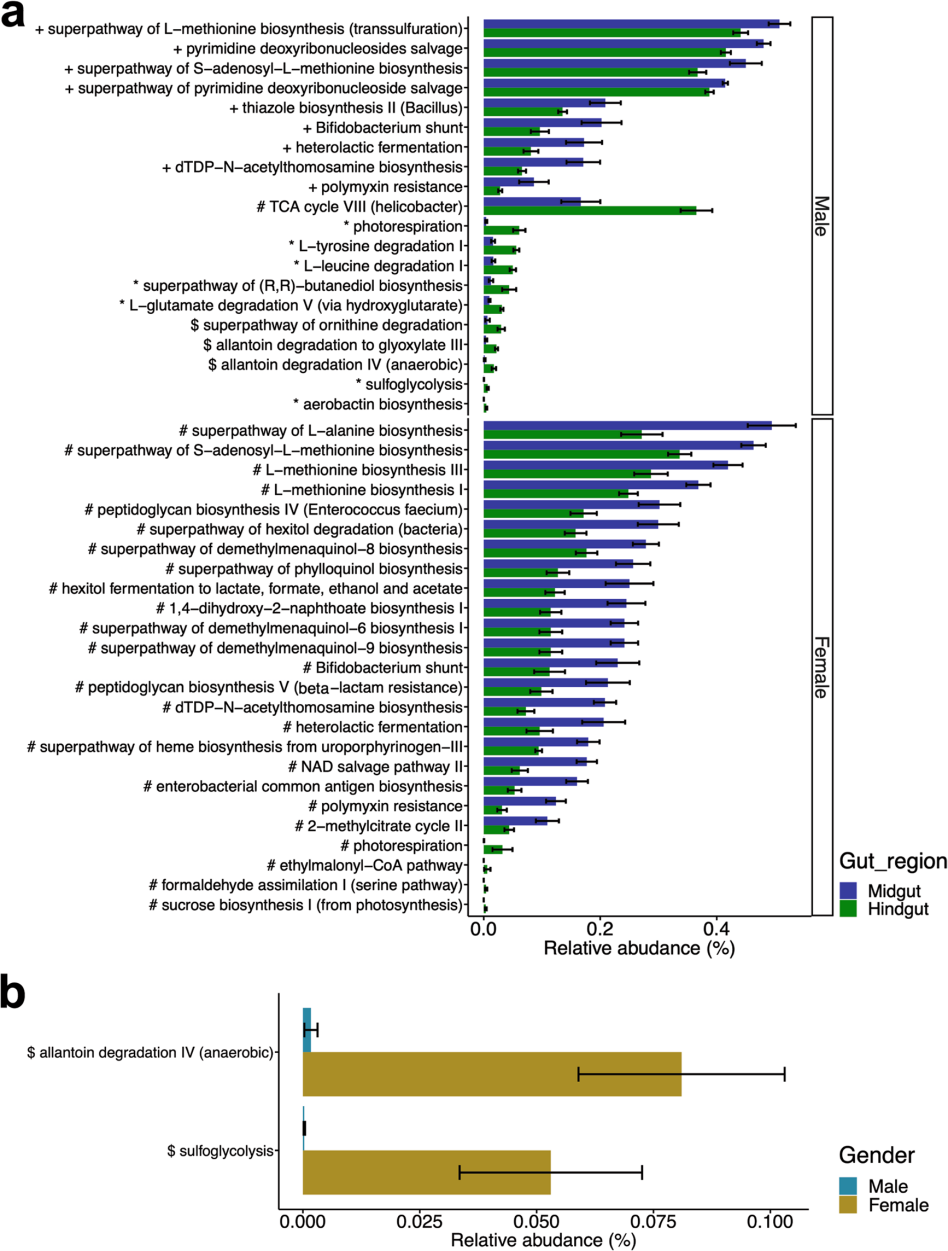
Predicted functional pathways differentially expressed between gut compartments and between genders. Error bars indicate standard error. Relative abundance of predicted functional pathways indicating significant variation between **a** gut compartments and **b** sexes. The mark before the pathway name indicates the combination of statistical tools that detected a significant difference (*all statistical tools, #ANCOMBC and DESeq2, $ALDEx2 and ANCOMBC, +ALDEx2 and DESeq2)

Marked differences were observed between the midgut and hindgut in the predicted pathways involved in amino acid turnover and metabolism. Overall, 20 and 25 pathways between the midgut and hindgut were significantly different in males and females, respectively, using at least two tools (Fig. 6a). The five pathways were predicted to be more abundant in the midgut than in the hindgut in both males and females, including the superpathway of S-adenosyl-L-methionine biosynthesis, bifidobacterium shunt, and heterolactic fermentation. Moreover, we found that amino acid biosynthesis (L-methionine and L-alanine) was enriched in the midgut. Notably, the hindgut contains abundant pathways involved in the TCA cycle (TCA cycle VIII and ethylmalonyl-CoA pathway) and amino acid degradation (L-tyrosine, L-leucine, L-glutamine, and ornithine degradation). Overall, these results suggest that the midgut showed significant enrichment of metabolic pathways related to amino acid synthesis and anaerobic metabolism (fermentation) in both sexes. In contrast, in the hindgut, significantly abundant metabolic pathways, such as the aerobic TCA cycle and ethylmalonyl-CoA pathway, might be involved in amino acid degradation and acetyl CoA-mediated processes.

In the analysis between the sexes, fewer pathways were significantly altered than those between the gut compartments. In the midgut, two pathways changed significantly, as determined by at least two methods: allantoin degradation IV (anaerobic) and sulfoglycolysis (Fig. 6b). For pathways with relative abundances greater than 1%, results from ANCOMBC alone showed that fatty acid salvage and glycolysis were significantly more abundant in the female midgut. In the DESeq2 results, pathways related to amine and polyamine degradation (aminosaccharides) were significantly more abundant in the female midgut (padj < 0.05). Additionally, the results with DESeq2 alone revealed that pathways related to sucrose degradation and peptidoglycan maturation were significantly enriched in the hindgut of females (padj < 0.05).

## Discussion

Crickets have emerged as an important insect model in biology, owing to their body size control, circadian rhythm, memory formation, regeneration mechanisms, and mating biology [19]. Given that gut bacterial communities play a significant role in host physiology, knowledge of the structure and sex-biased variation of microbial communities will greatly contribute to our understanding of the complex and dynamic interactions between the host and microbiome. To the best of our knowledge, the present study is the first to report a comparative analysis of bacterial communities associated with different subregions or sexes of crickets. The findings in the present study were solely based on the abundance and linked functions of 16S rRNA genes. We are thus cautious in accepting the following interpretation, and the fact that we did not quantify gene expression or microbial products.

The indices of species richness and diversity were highest in the hindgut, followed by the midgut and foregut in both females and males. Alpha and beta diversity indices among the gut compartments were significantly different. The ileum of crickets has feather-like bristles derived from the cuticle on the wall, providing a favorable environment in which most of the gut bacterial mass is held [45]. Malpighian tubules, in the anterior portion of the hindgut, excrete nitrogenous waste products, including uric acid and other solutes into the hindgut, thereby providing abundant nutrients for the gut bacterial population [6]. Moreover, the foregut compartment in *A. domesticus* is the most acidic [46]. An acidic foregut may be an unsuitable environment for the growth of gut bacteria. These physiological differences in the gut compartments may reflect differences in alpha diversity. For comparisons between the sexes, we found significantly higher diversity in males than in females in terms of Chao1 and Faith’s PD in the foregut and hindgut. A previous study on the gut microbiota of six grasshopper species also reported greater alpha diversity in males [47]. Nevertheless, in the differential relative abundance test, we found no significantly abundant bacterial taxa in the male hindgut. This discrepancy could be attributed to slight variations in the composition of the predominant bacterial species or fluctuations in rare species. Consistently, a study reported differentially abundant rare taxa in some species of butterflies, even though there was no significant difference in alpha diversity between sexes [48].

A comparison between the midgut and hindgut revealed distinct functional enrichment of bacterial species in each region. For example, *Apibacter*, belonging to the Weeksellaceae family, is abundant in the hindgut of both sexes. *Apibacter* has been reported to possess genes involved in respiratory nitrate reduction [49]. The large number of *Apibacter* in the hindgut may be reflected by the large amount of nutritive nitrogenous waste excreted by Malpighian tubes, providing a suitable environment for *Apibacter* specializing in nitrogen metabolism. Functional predictions also revealed that allantoin degradation was higher in the hindgut than in the midgut of males, suggesting the generation of ammonium, a product of allantoin degradation, in the hindgut. The resulting ammonium can be nitrified to nitrite and nitrate, as exemplified in studies using termites and beetles [50, 51], where Enterobacteriaceae present in the cricket guts might be responsible for nitrification activities to provide a source of nitrate respiration by *Apibacter* [52]. Collectively, these findings indicate nitrogen recycling in specific regions of the cricket gut.

Anaerobic environments in the midgut have been reported in various insects [53, 54]. We found that the genus *Bacteroides* was more abundant in the midgut than in the hindgut, which may be because of its biased anaerobic property. Furthermore, functional prediction revealed that anaerobic metabolism (fermentation) in both sexes and synthesis of menaquinone in females were more concentrated in the midgut. Menaquinones play an important role in electron transport in anaerobically respiring bacteria [55]. In contrast, the male hindgut was enriched in the TCA cycle, which suggests an aerobic environment in the hindgut. These findings support the hypothesis that the presence of bacteria and metabolism are dependent on varying oxygen levels in the gut, as reported in previous literature [56, 57].

We also found differential roles of the midgut and hindgut in amino acid turnover. There was an enriched function related to the degradation of amino acids (L-tyrosine, L-leucine, L-glutamine, and ornithine) in the hindgut compared to the midgut in males. Contrastingly, we observed that the amino acid (L-methionine, L-alanine) and coenzyme (S-adenosyl-L-methionine, thiazole biosynthesis II, menaquinone, and phylloquinone biosynthesis) biosynthesis pathways were abundant in the cricket midgut. Thus, cricket midgut bacteria may supplement the host’s amino acid and vitamin nutrition, as exemplified by a wide range of animals, such as aphids and beetles [58, 59]. Overall, amino acid and coenzyme biosynthesis pathways were abundant in the midgut of crickets, whereas amino acid degradation pathways were abundant in the hindgut.

Comparisons between sexes detected bacteria that could contribute to female physiology. Enterobacteriaceae was significantly abundant in the midgut of females. Enterobacteriaceae are involved in the metabolism of cellulose, xylan, pectin, and starch [60]. Nitrogen-fixing bacterial species belonging to the Enterobacteriaceae family may assist in supplying nitrogen to satisfy female-specific nutritional needs such as egg production [61]. Pseudomonadaceae was also detected in females as a family with a relative abundance > 1%. Pseudomonadaceae has been demonstrated to aid in host digestion and increase the utilization of nutrients in *Dendroctonus rhizophagus* (Coleoptera: Curculionidae) [62]. Enterobacteriaceae and Pseudomonadaceae have been reported to improve egg production in the olive fly *Bactrocera oleae* (Diptera: Tephritidae) by utilizing available nitrogen compounds to supply the missing amino acids for the host or by being absorbed into the host as a source of amino acids [2, 63]. These previous studies support our finding that Enterobacteriaceae and Pseudomonadaceae are significantly abundant in the female gut. Significant enrichment of allantoin degradation was detected in the midgut of females compared to that of males using two statistical tools. In the midgut of larvae of *Anoplophora glabripennis* (Coleoptera: Cerambycidae), nitrogen is presumed to be recycled by the allantoin degradation pathway [64]. In addition, gut bacteria have been reported to promote egg production in olive fruit flies by recycling nitrogen via the metabolism of nitrogenous waste metabolites [2]. In the present study, we found that glycolytic degradation was enriched in the female midgut. In the cotton leafworm, *Spodoptera littoralis* (Lepidoptera: Noctuidae), the adult female microbiome has more genes for energy metabolism than the male microbiome [65]. Furthermore, we found that the ratio of Firmicutes to Bacteroidota was significantly higher in females. A higher abundance of Firmicutes over Bacteroidota has been reported to increase the ability to harvest energy and supply nutrients [41]. These findings suggest that female midgut bacteria may contribute to the promotion of egg production as well as to efficient nutrient utilization, although the mechanisms driving sexually divergent gut bacteria remain unknown.

The present study provides insights into the role of the gut compartments and sex in host-gut bacterial interactions. However, this study was conducted using 16S rRNA amplicon analysis. Therefore, we were not able to discuss the cricket gut microbiota at the species level and gene contents coded in the whole genome of bacteria. Detailed gut microbial analysis using metagenomic shotgun sequencing and meta-transcriptomic analysis will be required in the future.

### Supplementary Information

Fig. S1 Rarefaction curves based on the number of ASVs of the foregut, midgut, hindgut of both male and female. (PNG 387 kb)

High resolution image (EPS 736 kb)

Fig. S2 Bacterial composition of the feed. Relative abundance of bacterial communities at (**a**) the phylum level, (**b**) the family level, and (**c**) the genus level. (PNG 273 kb)

High resolution image (EPS 732 kb)

(PNG 273 kb)

High resolution image (EPS 732 kb)

Fig. S3 Alpha diversity indices of gut bacterial communities, including Chao1, Shannon, Simpson, and Faith’s phylogenetic diversity (PD) with singletons removed. The Wilcoxon rank sum test was used to compare the difference in the gut compartments. Single asterisk indicates *P* < 0.05. Double asterisk indicates *P* < 0.01. (PNG 188 kb)

High resolution image (EPS 459 kb)

Table S1 Number of raw and quality filtered reads for both males and females per sample. (XLSX 12 kb)

Table S2 Relative abundance and mean relative abundance tables for males and females at the phylum, family, and genus level. (XLSX 141 kb)

Table S3 Raw data and test results for alpha diversity. (XLSX 19 kb)

Table S4 Weighted and unweighted UniFrac raw data and PERMANOVA test results. (XLSX 62 kb)

Table S5 Results of differential relative abundance analysis in DESeq2. (XLSX 25 kb)

Table S6 Results of differential relative abundance analysis in ALDEx2. (XLSX 26 kb)

Table S7 Results of differential relative abundance analysis in ANCOMBC. (XLSX 32 kb)

Table S8 Raw data and test results for alpha diversity with singletons removed. (XLSX 18 kb)

## Data Availability

All data generated or analyzed during this study are included in this published article and its supplementary information files. Raw sequence data were deposited in the NCBI BioProject (PRJNA933524), BioSample (SAMN33267117-SAMN33267143, SAMN33267295-SAMN33267309), and SRA (SRR23435345-SRR23435359, SRR23435289-SRR23435315).
