# Supplementary figures and images for: Spatial and Sexual Divergence of Gut Bacterial Communities in Field Cricket Teleogryllus occipitalis (Orthoptera: Gryllidae)
Source: Microb Ecol. 2023 Jul 21;86(4):2627–41. doi: 10.1007/s00248-023-02265-z (PMC10640434; doi:10.1007/s00248-023-02265-z)

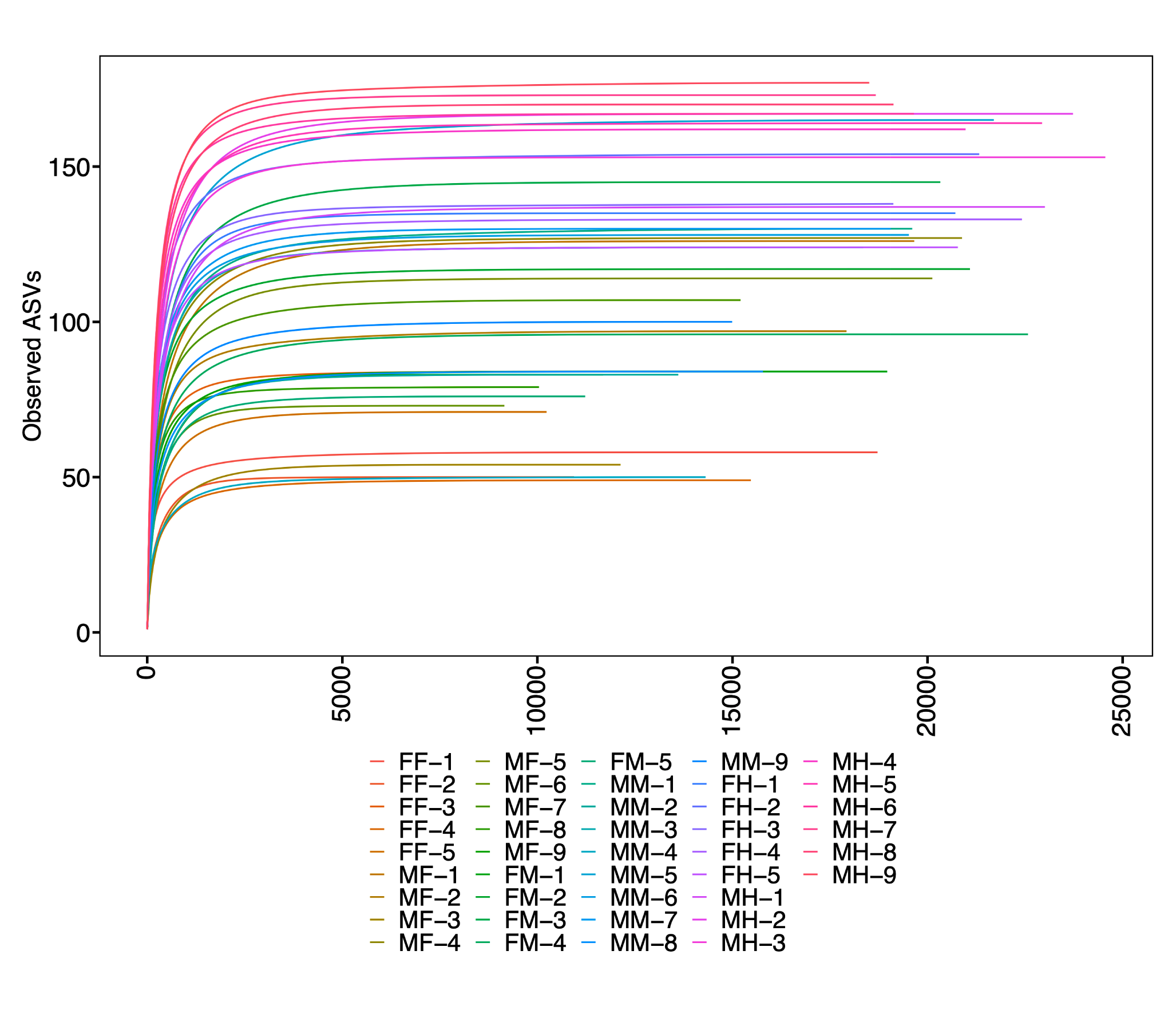

Supplement: Supplementary file 1 — Rarefaction curves based on the number of ASVs of the foregut, midgut, hindgut of both male and female. (PNG 387 kb) [file 248_2023_2265_Fig7_ESM.png]

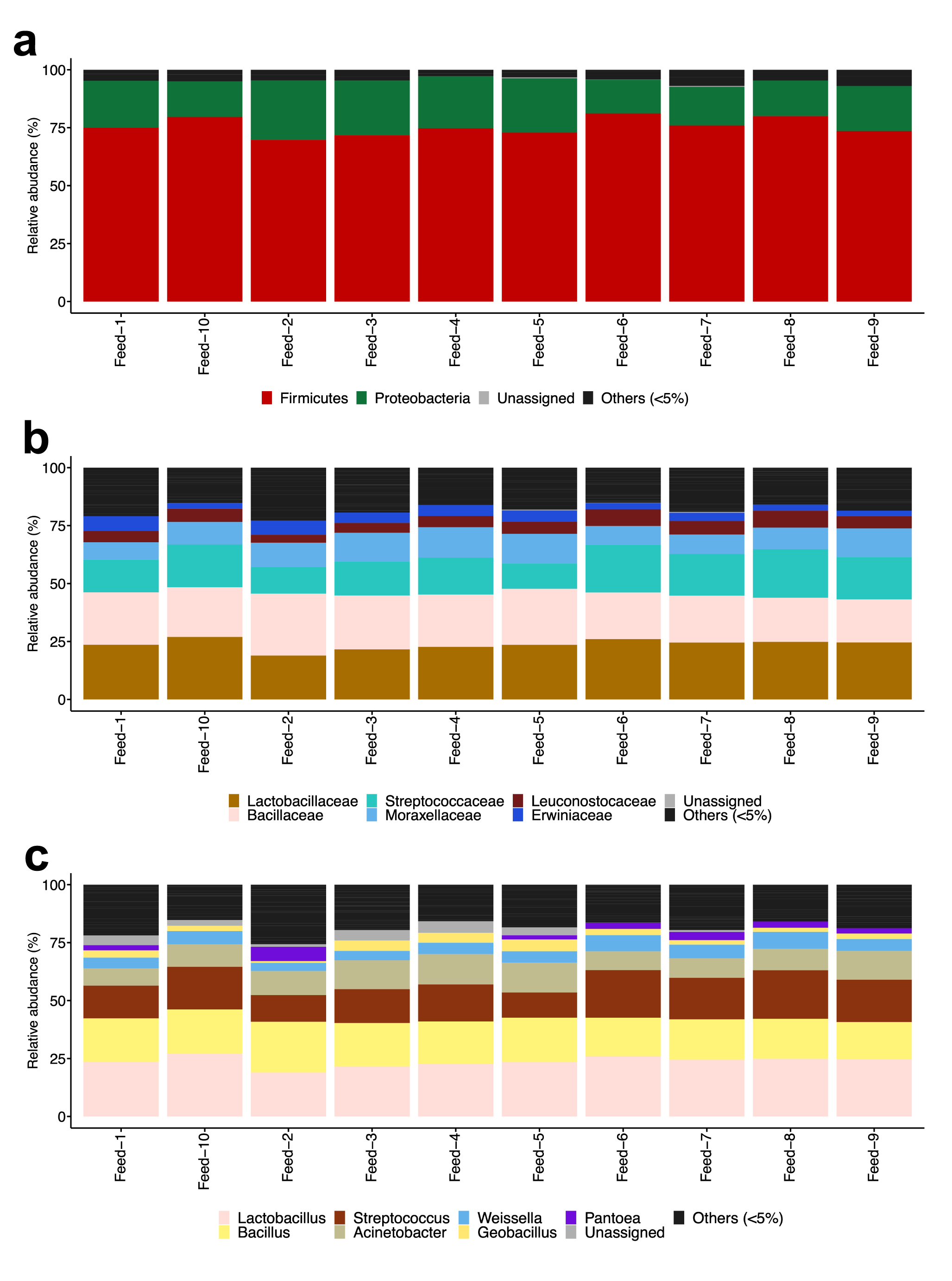

Supplement: Supplementary file 3 — Bacterial composition of the feed. Relative abundance of bacterial communities at (a) the phylum level, (b) the family level, and (c) the genus level. (PNG 273 kb) [file 248_2023_2265_Fig8_ESM.png]

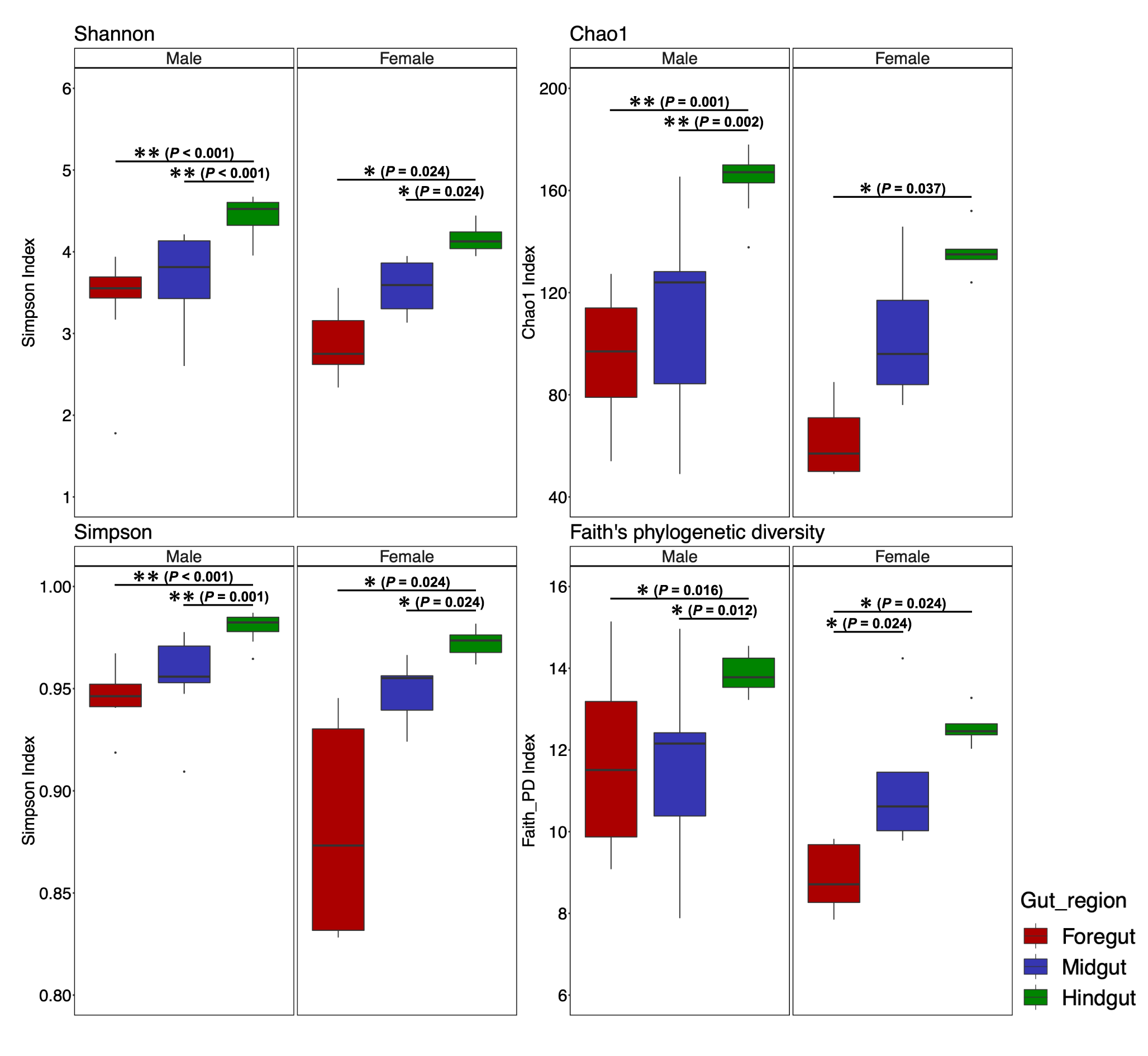

Supplement: Supplementary file 7 — Alpha diversity indices of gut bacterial communities, including Chao1, Shannon, Simpson, and Faith’s phylogenetic diversity (PD) with singletons removed. The Wilcoxon rank sum test was used to compare the difference in the gut compartments. Single asterisk indicates P < 0.05. Double asterisk indicates P < 0.01. (PNG 188 kb) [file 248_2023_2265_Fig10_ESM.png]
